# The role of protective liners and glass ionomer in managing pulp temperature during light curing 

**DOI:** 10.4317/jced.61703

**Published:** 2024-06-01

**Authors:** Vanessa-Dias-Barboza Munhoz, Mateus-Garcia Rocha, Americo-Bortolazzo Correr, Mario-Alexandre-Coelho Sinhoreti, Saulo Geraldeli, Dayane Oliveira

**Affiliations:** 1 Department of Restorative Dentistry, Piracicaba Dental School, University of Campinas, Piracicaba, SP, Brazil; 2 Department of Restorative Dental Science, College of Dentistry, University of Florida, Gainesville, FL, USA; 3 Division of Biomedical Materials, School of Dental Medicine, East Carolina University, Greenville, NC, USA

## Abstract

**Background:**

To evaluate the thermal insulation of protective liners and glass ionomer cement during light-curing procedures.

**Material and Methods:**

Human third molars underwent Class I preparations with dimensions 5 mm long × 4 mm wide × 4 mm deep in a standardized manner ensured a consistent ±0.5 mm dentin thickness at the pulpal floor. The teeth were attached to a customized oral cavity chamber simulator with a circulating bath at a standardized temperature of 34.2 ± 1oC. The temperature variations at the pulpal floor were captured in real-time by video using an infrared thermal camera (FLIR ONE Pro, FLIR Systems). The materials evaluated were: Dycal (Dentsply), TheraCal LC (Bisco), Activa (Pulpdent), and Fuji II LC (GC). All light-activation procedures were performed with the same light-curing unit (Valo Grand, Ultradent) in standard mode, 1000 mW/cm2, and time of exposure following manufacturer instructions. A power analysis was conducted to determine the sample size considering a minimal power of 0.8, with α=0.05. Statistical analyses were performed using ANOVA and Tukey’s test for multiple comparisons.

**Results:**

The temperature at the pulpal floor increased above the 5.5 ºC safety threshold difference for clinical scenarios tested. None of the materials provided proper thermal insulation for light-curing procedures (*p* = 0.25). The higher the number of light-cured steps, the longer the pulp remained above the 5.5 ºC temperature threshold.

**Conclusions:**

The materials tested provided improper thermal insulation (Δ > 5.5 ºC). Thus, prolonged or multiple light-curing exposures can be harmful to the pulp tissues. Therefore, for indirect pulpal capping procedures, self-cured materials or a reduced number of steps requiring light curing must be adopted to reduce the amount of time the pulp remains above the 5.5 ºC safety temperature threshold.

** Key words:**Dental Pulp Capping, Calcium hydroxide, Bioactive, Thermal Damage.

## Introduction

Concerns regarding the impact of temperature increases on the dental pulp have been a longstanding issue in dentistry. These concerns range from the heat generated during cavity preparations to heat associated with light-curing procedures (1,2). In the latter, the visible light triggers a quantum transition in the material’s electrons, leading to a vibrational excited state. This state causes the atoms to collide and dissipate the energy in the form of heat (3). This generated heat can then transfer from the material to the tooth, posing potential risks, specifically to the pulp (4,5).

Due to its composition, morphology, and properties, the dentin serves as a protective barrier against thermal damage (6). However, in cases of deep preprations where only a thin layer of dentin remains, there is an elevated risk of thermal injury to the pulp (7). For this reason, it is recommended that liners and bases possess insulation properties similar to those of the the dentin they are intended to replace. These properties include low thermal conductivity and thermal insulation against heat (8,9).

For many years, calcium hydroxide cements have been considered the gold standard for pulp protection (10). These cements consist of a chemically cured dual paste system with a basic pH. The high pH makes these cements highly cytotoxic, immediately causing necrosis in tissues in direct contact and eliciting an inflammatory reaction. Despite these cytotoxic effects, this process stimulates the deposition of a reactive dentin bridge, which serves as a positive outcome (11,12). On the other hand, these cements are highly soluble and lack adhesive properties (13). To overcome these disadvantages, a light-cured resin-modified calcium silicate-based material, TheraCal LC (Bisco), was developed. This material features reduced solubility and enhanced adhesive and mechanical properties (14).

Resin-modified glass ionomer cements are also indicated for deep tooth-preparations. These materials exhibit both chemical bonding to dental tissues and the ability to release fluoride while offering mechanical properties suitable for their use as a restorative material (15,16). A particular glass ionomer material, Activa (Pulpdent), described by its manufacturer as a “light-cured resin-modified calcium silicate”, was designed to serve as an insulating layer for thermal protection in deep preparations while being used as a dental filling that can be applied in 4 mm thick increments (17,18).

In solid materials such as dental liners, heat is transferred through conduction, moving from one particle to another particle (19). The varied compositions of existing materials used as liners in dentistry suggest they might have different thermal properties, particularly regarding their effectiveness in providing thermal insulation. However, this aspect has received limited attention in dentistry. Therefore, this study focused on accessing the thermal insulation of different protective liners and glass-ionomer cements during light-curing procedures. The tested hypotheses of this study were: 1) protective liners would be effective in providing thermal insulation during light-curing procedures; and 2) base materials would be effective in providing thermal insulation during light-curing procedures.

## Material and Methods

The Health Center Institutional Review Board of the University of Florida approved this study (IRB202201548).

-Teeth preparation 

First, all human third molars extracted were submitted to an occlusal surface flattening using a polishing machine (AUTOMET 250, Buehler, Lake Bluff, IL, USA). Then, their roots were sectioned in the dental-enamel junction using a cutting machine (ISOMET 1000- Buehler Ltd., Lake Bluff, IL, USA). This preparation allowed for the subsequent measurement of the pulpal floor thickness, as further described.

Class I preparations measuring 5 mm long × 4 mm wide and 4 mm deep were created using a rotatory hand drill (Kavo Dental, Charlotte, NC, USA) and cylindrical diamond burs with the aid of a preparation standardizing machine (Odeme Dental Research, Pompano Beach, FL, USA). After the preparation, the pulpal floor thickness was measured with a dental caliper to ensure a remaining dentin thickness of ±0.5 mm. For standardization purposes, the light-transmittance through the pulpal floor of the Class I preparations was evaluated to randomize the samples (see light-transmittance standardization analysis section).

-Light-curing unit characterization 

The mean radiant emittance (mW/cm2) for the light-curing unit used in the study (VALO Grand, Ultradent, South Jordan, UT, USA) was measured using a spectrometer-based instrument (MARC Resin Calibrator, BlueLight Analytics, Nova Scotia, Canada). The mean radiant emittance used in the study was 1009 ± 11.86 mW/cm2.

-Light-transmittance standardization analysis 

The light-transmittance through pulpal floor of the Class I preparations were recorded with a spectrophotometer (MARC Resin Calibrator, BlueLight Analytics, Nova Scotia, Canada). Each tooth was positioned above the input sensor of the spectrophotometer and, the light-transmittance (mW/cm2) was recorded. The light-transmittance results were used to randomize the samples. The randomization was statically evaluated using one-way ANOVA. The randomization distribution passed the Shapiro-Wilk normality test (W > 0.8; *P* > 0.6) and three different variance analysis tests, the ANOVA, the Brown-Forsythe test, and the Bartlet’s test (*P* > 0.9; R2 < 0.05).

-Temperature variations at the pulpal floor analysis 

**Figure 1:**
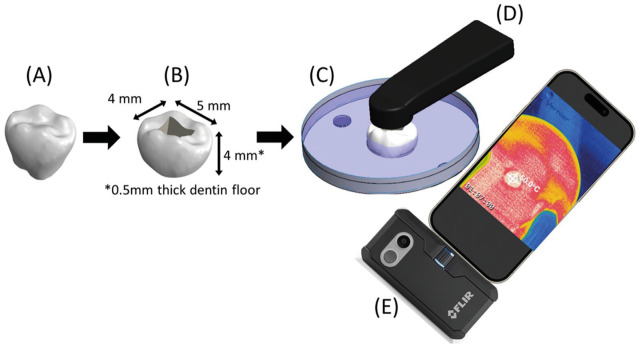
Customized simulated oral cavity chamber: (A) Extracted third molar; (B) Class I preparation; (C) Circulating water bath with temperature digitally controlled; (D) curing light; (E) infrared thermal camera positioned below the pulp chamber to capture temperature variations by video in real-time, with a standardized fixed distance.

To better simulate clinical conditions, the teeth containing the Class I preparations were placed in an oral cavity chamber simulator, customized with a circulating water bath with the temperature being digitally controlled at 34.2ºC (± 1ºC). The specimenss were fixed with wax in the simulator with their occlusal surface facing upwards while leaving the pulpal floor uncovered and oriented towards the bottom, where an infrared camera (FLIR ONE PRO, FLIR Systems, Wilsonville, OR, USA) was positioned immediately underneath to live record temperature variations (oC) in the pulp chamber during the light-activation processes, with a standardized fixed distance, as depicted in Figure 1.

The infrared camera used had a thermal sensitivity of 150 mK, or <0.10 ºC. The video database of each specimen was then transferred into a data sheet using the camera’s software (FLIR Tools, FLIR Systems, Wilsonville, OR, USA).

-Pulp capping procedures 

The materials evaluated were: TheraCal (Bisco, Schaumburg, IL, USA), Dycal (Dentsply, Charlotte, NC, USA), Activa (Pulpdent, Watertown, MA, USA), and Fuji II LC (GC, Luzern, Switzerland).

Activa: First, a single coat of universal adhesive (Adhese Universal, Ivoclar Vivadent, Schaan, Liechtenstein) was applied with a micro brush for 20 seconds, air-dried for 5 seconds, and light-cured for 10 seconds. Activa was then dispensed in the Class I preparation in one layer that was 4-mm thick and light-cured for 20 seconds. As recommended by the manufacturer, the first 2 mm of the mixed material was discarded prior to utilization.

TheraCal: TheraCal was directly dispensed in the Class I preparation in a single layer that was less than 1 mm thick and light-cured for 20 seconds. The restoration process continued with Fuji II LC. Thus, its cavity conditioner was applied as recommended by the manufacturer and then rinsed and dried accordingly. Then, the Fuji II LC capsule was activated and mixed according to the manufacturer’s instructions before being applied over the TheraCal layer in two layers that were 2-mm thick and individually light-cured for 20 seconds.

Dycal: Equal volumes of both the base and catalyst pastes were dispensed onto a mixing pad and thoroughly mixed until a homogeneous color was achieved. The material was then applied to the pulpal floor in a thin layer of less than 1 mm thick using a Dycal applicator. After allowing a setting time of 3 minutes, the restoration process continued with Fuji II LC following the same steps as described above.

Fuji II LC: In this scenario, the restoration process included only the use of Fuji ll LC as described above with no liner application.

-Statistical Analysis 

A power analysis was conducted to determine the sample size to provide a power of at least 0.8 with α=0.05 (β=0.2). Data normality and homoscedasticity were accomplished with Shapiro-Wilk and Levine’s test, respectively. Statistical analyses were performed using ANOVA and Tukey’s test for multiple comparisons.

## Results

**Figure 2:**
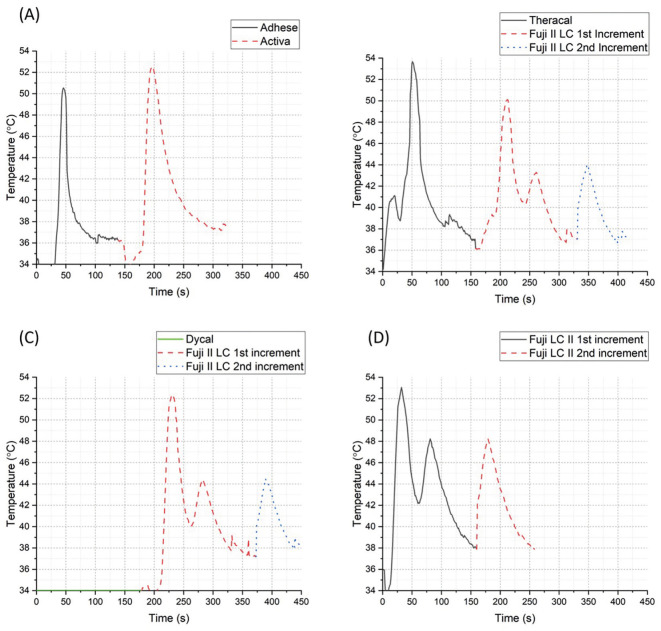
Depiction of variations at the pulpal floor when restoring with (A) Activa (Pulpdent); (B) TheraCal (Bisco) and Fuji II LC (GC); (C) Dycal (Dentsply) and Fuji II LC (GC); (D) Fuji II LC (GC).

Figure 2 depicts an overview of the temperature variations at the pulpal floor associated with different restorative scenarios tested in this study.  Table 1 describes the peak temperatures achieved within each light-curing step performed in each restorative scenario. As can be observed in  Table 1, there were no statistically significant differences between the restorative scenarios with or without the application of a liner (*p*=0.25). All restorative scenarios increased the pulpal floor temperature from the 34°C baseline over the 42.5°C safety baseline (5.5°C safety threshold). Even when Fuji II LC was applied as a second layer, the temperature increased above the 42.5°C threshold.

## Discussion

Restorative procedures in deep preparations require particular measures, especially to prevent pulp damage from heat generation. Based on the findings of this study, the first tested hypothesis that protective liners are effective in providing thermal insulation during light-curing procedures was rejected. In all restorative scenarios tested, the temperatures at the pulpal floor exceeded the critical threshold of 42.5ºC, commonly considered the maximum temperature the pulp can tolerate without resulting in irreversible biological damages, such as necrosis and irreversible pulpitis (6,20,21).

Liners used to be traditionally considered suitable materials for protecting the pulp from heat damage based on their lower thermal conductivity, which is even lower than those of dentin tissue (8). However, it is important to highlight that this information is outdated due to two primary reasons. Firstly, light-curing procedures were not commonly or necessarily employed during restorative procedures. Thus, concerns regarding heat generation were primarily focused on tooth preparation. Secondly, the curing lights available at that time, when applied to restorative procedures, were not the high-power LEDs that are used nowadays. According to the findings of this study, the lower thermal conductivity from these liners alone does not ensure that these liners can effectively serve as thermal insulators. While having a low thermal conductivity suggests that the material will not readily conduct thermal energy, it does not necessarily imply that it can offer adequate thermal insulation against temperature rise during light-curing procedures (3).

It is possible to consider that the temperatures reached during the light-curing process are not the ideal scenarios where the liners could act as thermal insulators since thermal conductivity is a temperature-dependent thermo-physical property, with respect to which each class of materials behaves differently (22,23). Therefore, when the temperature of the insulator material increases, its thermal resistance decreases, as if its insulating ability were partially lost (24). That way, ignoring the temperature dependency of the material’s thermal conductivity property can cause under- or over-estimations of its behavior in front of heat flow (25). Thus, while previous studies have suggested that the low thermal conductivity of liners might protect the pulp against minor temperature fluctuations, such as those caused by hot or cold intake (8), this study has demonstrated that their thermal properties are insufficient for effectively insulating against the level of heat generated by light-curing procedures.

On the other hand, in terms of critical temperature duration, the use of the light-cured liner, TheraCal, increased the overall time that the pulp remained above the 42.5ºC threshold, thereby increasing the risk of necrosis (21). Thus, chemically cured liners such as the calcium hydroxide cement could avoid an additional light-curing step, as well as an additional increase in the overall time that the pulp would remain above the 42.5ºC safety threshold. This is particularly important to take into consideration in light of the fact that studies indicate no statistically significant difference in the clinical success between Dycal and TheraCal for indirect pulp-capping procedures (10).

The second hypothesis that glass-ionomer materials are effective in providing thermal insulation during light-curing procedures was also rejected. There were no differences in the peak temperature achieved between the different restorative scenarios tested. According to the results of this study, statistical differences were observed between the Fuji II LC first and second increments. This outcome was anticipated since the thickness of the material does not alter its thermal conductivity, but it does increase its thermal resistance, thereby mitigating heat flow (26). On the other hand, this increase in thermal resistance was still not enough to prevent the temperature from rising above the critical safety threshold of 42.5ºC.

Throughout the entire restorative procedure, every scenario examined in this study involved subjecting the pulp to at least 40 seconds of thermal damage. An earlier ex-vivo experiment showed that a temperature increases of 5.5°C or higher for 40 seconds can lead to pulpal death (2). Considering the possibilities to avoid pulp damage, clinicians should consider choosing and using materials in a way that minimizes the number of light-curing procedures. Alternatives could include the use of chemically cured materials, as well as bulk-cured materials, to reduce the number of light-curing steps required with light-cured materials (27).

Studies show that Activa can be used in a single bulk-fill increment and still exhibits comparable or superior physical and mechanical properties to other similar glass ionomer cements (18,28). This attribute can expedite the operative procedure, in addition to minimizing the number of light-curing steps when compared to other glass ionomer cements. However, it is important to point out that the use of an adhesive system is recommended to improve bond strength when using Activa as the restorative material (29). In this case, its indication over other materials can be questionable depending on the depth of the preparation being filled and the number of increments each restorative technique would require light activation.

To summarize, the practice of relying solely on thermal conductivity to assess the insulating capacity of a dental liner was challenged in this study. The heat transfer within a material is influenced not just by its thermal conductivity but also by its heat capacity, both of which are temperature-dependent properties crucial for thermal insulation (30,31). Moving forward, the development of new liners and base materials should consider both thermal conductivity and heat capacity, especially in light of the temperature ranges experienced during photoactivation with high-power LEDs, to ensure adequate insulating properties, taking into account the temperature dependence of these thermal characteristics. Moreover, in future studies, thermal conductivity and heat capacity should both be considered in a specific temperature range to achieve proper thermal insulation on liners and restorative dental materials.

## Conclusions

Within the limitations of this *in vitro* study, it was possible to conclude the protective liners and glass ionomer materials tested provide improper thermal insulation (Δ > 5.5 ºC). Thus, prolonged or multiple light-activation exposures can be harmful to the pulp tissues. Therefore, for indirect pulp capping procedures, self-cured materials or a reduced number of steps requiring light activation must be adopted to reduce the amount of time the pulp remains above the 5.5ºC safety temperature threshold.

## Figures and Tables

**Table 1:** Temperature variations at the pulpal floor when restoring with Activa (Pulpdent); TheraCal (Bisco) and Fuji II LC (GC); Dycal (Dentsply) and Fuji II LC (GC); Fuji II LC (GC).

Group	Peak temperature ºC			
	Initial temperature	First light-activation	Second light-activation	Third light-activation
Activa	34.4 (± 0,78)	Adhesive (20 s) 50,54 (± 1,43) A	Activa (20s) 52,59 (± 1,84) A	-
TheraCal and Fuji II LC	34.11 (± 0,34)	Theracal (20s) 53,66 (± 0,73) A	Fuji II LC (20 s) 50,11 (± 2,39) AB	Fuji II LC (20 s) 44,04 (± 3,52) C
Dycal and Fuji II LC	34.27 (± 0,99)	Fuji II LC (20 s) 52,41 (± 1,43) A	Fuji II LC (20 s) 44,48 (± 2,13) C	-
Fuji II LC	34.74 (± 1,09)	Fuji II LC (20 s) 53,06 (± 3,32) A	Fuji II LC (20 s) 46,58 (± 2,34) BC	-

Capital letters indicate significant differences between different materials tested.

## Data Availability

The datasets used and/or analyzed during the current study are available from the corresponding author.
